# Knowledge, attitudes, practices, and barriers toward drug–drug interaction management among pharmacists in Riyadh, Saudi Arabia: a cross-sectional study

**DOI:** 10.3389/fpubh.2026.1727731

**Published:** 2026-03-13

**Authors:** Othman AlOmeir, Abdullah Khatim R. Alanazi, Mulham Ahmed Alnhas, Mansour Ahmed Ajarem, Zakwan Jamilurehman Hafiz, Syed AbdulMuqeet, Seham Muqbil Alanazi, Majed Sadun Alshammari, Hessah khaled ALjanobi, Hamoud Alotaibi, Deemah Alhamad, Nada Aldhahri, Majed A. AlJuaie, Syed Mohammed Basheeruddin Asdaq

**Affiliations:** 1 Department of Clinical Pharmacy, College of Pharmacy, Shaqra University, Shaqra, Saudi Arabia; 2 Department of Pharmacy Practice, College of Pharmacy, AlMaarefa University, Riyadh, Saudi Arabia; 3 Assistant Consultant, King Abdulaziz Medical City, Riyadh, Saudi Arabia; 4 Department of Pharmacy, King Abdulaziz Medical City, Riyadh, Saudi Arabia; 5 Department of Nursing, King Abdulaziz Medical City, Riyadh, Saudi Arabia; 6 Department of Pharmacy, King Saud University Medical City, Riyadh, Saudi Arabia; 7 Department of Pharmacy, King Khaled Eye Specialist Hospital and Research Center, Riyadh, Saudi Arabia; 8 Research Center, Deanship of Scientific Research and Postgraduate Studies, AlMaarefa University, Riyadh, Saudi Arabia

**Keywords:** attitudes, barriers, drug–drug interactions, knowledge, pharmacists, practice, Saudi Arabia

## Abstract

**Background:**

Drug–drug interactions (DDIs) pose a serious challenge in pharmaceutical care, affecting medication safety and treatment outcomes. Pharmacists play a crucial role in identifying, preventing, and managing DDIs. This study aimed to evaluate the knowledge, attitudes, practices, and perceived barriers related to DDIs among pharmacists in Riyadh, Saudi Arabia, between September and December 2024.

**Methods:**

A cross-sectional survey was conducted among pharmacists in Riyadh using a structured questionnaire to gather demographic data and assess knowledge, attitudes, practices, and perceived barriers toward DDI management. Statistical analyses, including correlation and binary logistic regression, were employed to investigate the associations.

**Results:**

Among the participants, 63.8% were 30–39 years (40.8%). A moderate positive correlation was found between attitudes and practices (*r* = 0.439, *P* < 0.001), suggesting that favorable attitudes were associated with better practices. Female pharmacists (*P* < 0.001, OR = 0.381) and those with formal DDI training (*P* < 0.001, OR = 5.467) reported significantly better practices. Perceived barriers were lower among younger pharmacists (*P* = 0.024, OR = 0.593), those with higher qualifications (*P* = 0.004, OR = 0.675), and those in specific work settings (*P* < 0.001, OR = 0.621), while more experienced pharmacists reported greater barriers (*P* < 0.001, OR = 1.933). Formal training did not significantly impact perceived barriers (*P* = 0.201).

**Conclusion:**

Formal training in DDIs markedly improves pharmacists' practices. Younger age and higher qualifications are associated with fewer perceived barriers. Tailored educational interventions are crucial to enhance DDI management and promote patient safety.

## Introduction

Drug–drug interactions (DDIs) occur when the concurrent administration of two or more medications alters their expected pharmacological effects, potentially leading to therapeutic failure or increased toxicity. This issue has emerged as a critical concern in clinical practice, particularly among patients with chronic conditions requiring polypharmacy (1–3). The clinical consequences of DDIs are substantial, ranging from mild adverse drug reactions to serious, life-threatening events and treatment failures (4–6).

DDIs may arise from both pharmacokinetic and pharmacodynamic mechanisms (7, 8). Pharmacokinetic interactions involve changes in drug absorption, distribution, metabolism, or excretion, while pharmacodynamic interactions result from altered pharmacological responses. For example, some medications can induce or inhibit hepatic enzymes like CYP450, significantly impacting drug concentrations and safety profiles (9, 10). Furthermore, patient-specific characteristics such as age, sex, comorbidities, genetic polymorphisms in drug-metabolizing enzymes, and differences in drug formulations contribute to the variability and severity of DDIs (11, 12).

Globally, DDIs are a leading cause of preventable adverse drug events (ADEs), increasing healthcare costs and compromising patient safety (4). It is estimated that DDIs account for approximately 5% of adverse events in hospitalized patients and up to 11% of prescriptions in outpatient care (13, 14). In Saudi Arabia, nearly 7% of hospital admissions linked to drug-related problems are associated with DDIs, with a disproportionate impact on older adults (15, 16). Older population are especially vulnerable due to age-related physiological changes and the frequent use of multiple medications. Studies have reported DDI prevalence rates among the older population ranging from 0.8 to 90.6%, reflecting wide variability across populations and healthcare settings (3). For instance, one Saudi study reported that 90.6% of older population patients in ambulatory care encountered at least one DDI (17). Another study indicated that around 20% of patients exposed to potential DDIs are at risk of experiencing clinically significant adverse reactions (18).

In Saudi Arabia, medication safety has become a national priority under the Kingdom's Vision 2030 initiative, which emphasizes healthcare transformation, patient safety, digital health integration, and quality improvement across healthcare institutions (19). The Health Sector Transformation Program aims to strengthen regulatory oversight, enhance pharmacovigilance systems, and promote evidence-based pharmaceutical care. Within this framework, the Saudi Food and Drug Authority (SFDA) plays a pivotal role in regulating medicines, monitoring adverse drug reactions, and implementing national pharmacovigilance programs to improve medication safety (20). The SFDA operates the National Pharmacovigilance Center and facilitates electronic reporting systems for adverse drug events to ensure safe prescribing and dispensing practices (20). Additionally, healthcare institutions across the Kingdom are increasingly adopting electronic prescribing systems and clinical decision support tools to detect and prevent potential DDIs at the point of care, aligning with national digital health transformation strategies (19).

The high prevalence and serious implications of DDIs demand robust strategies for prevention and management. These include the integration of clinical decision support tools, better patient record monitoring, and continuous professional education. Pharmacists, by virtue of their expertise in pharmacotherapy, are central to these efforts (21, 22). However, their ability to effectively manage DDIs is often hindered by insufficient knowledge, high workload, lack of access to drug interaction databases, and systemic barriers in both hospital and community settings (23).

Although several studies in Saudi Arabia have examined pharmacists' knowledge of drug–drug interactions (DDIs) (24, 25), the majority have been limited to community pharmacy settings. To date, only one study has investigated both attitudes and practices related to DDIs among pharmacists (23); however, it did not explore the perceived barriers that may hinder effective DDI management. Furthermore, key demographic and professional factors—such as sex, age, clinical experience, and formal training—that may significantly influence pharmacists' ability to manage DDIs remain insufficiently studied.

Given the evolving scope of pharmacists' responsibilities and their frontline role in ensuring medication safety, particularly within the context of national healthcare reforms and strengthened regulatory frameworks in Saudi Arabia, this study aimed to evaluate the knowledge, attitudes, and practices of pharmacists in Riyadh, Saudi Arabia, regarding DDIs. Furthermore, the study investigated the barriers impeding optimal DDI management and assessed the influence of formal training on improving outcomes.

## Subjects and methods

### Study design

A cross-sectional study design was employed to assess the knowledge, attitudes, practices, and perceived barriers related to drug–drug interactions (DDIs) among pharmacists in Riyadh, Saudi Arabia. This approach allowed for a comprehensive snapshot of current pharmacist competencies and systemic challenges in DDI management.

### Population and sampling

The target population for this study comprised licensed pharmacists working in diverse professional settings within Riyadh, including community pharmacies, hospitals, clinics, industrial sectors, and academic institutions. A stratified random sampling technique was employed to ensure adequate representation of participants from various practice environments and geographic regions within the city. This approach enhanced the generalizability of the findings across different pharmacy sectors.

Inclusion criteria

Participants were eligible for inclusion if they met the following criteria:

1) Licensed pharmacists currently practicing within Riyadh.
2) Aged 20 years or older.
3) Actively engaged in direct or indirect patient care activities.

Exclusion criteria

The following individuals were excluded from the study:

1) Non-pharmacist healthcare professionals.
2) Unlicensed pharmacists.
3) Individuals not currently practicing in any pharmacy-related role.

### Study instrument

A structured questionnaire was carefully developed to measure pharmacists' knowledge, attitudes, practices, and perceived barriers concerning DDI management. The questionnaire was designed in English based on a comprehensive review of existing literature (23–25) to ensure alignment with study objectives and current pharmacy practice in Saudi Arabia.

#### Questionnaire validation

Content Validity: An expert panel of six professionals from pharmacology, clinical pharmacy, and public health reviewed the draft questionnaire. Based on their feedback, refinements were made to improve question clarity, item relevance, and structural coherence, ensuring comprehensive coverage of DDI-related competencies.

Criterion validity: the questionnaire content was benchmarked against previously validated instruments and peer-reviewed studies that focused on pharmacists' abilities to identify and prevent DDIs. This ensured alignment with recognized standards and best practices.

Construct validity: exploratory factor analysis was conducted to evaluate the theoretical coherence and internal structure of the questionnaire. This analysis confirmed that the questionnaire domains—knowledge, attitudes, and practices—were distinct and well-defined.

Internal Consistency: reliability testing using Cronbach's alpha produced a score of 0.831, indicating high internal consistency across questionnaire domains and supporting the tool's reliability for research purposes.

Face validity: a pilot test involving 20 pharmacists from the target population was carried out to assess the clarity, relevance, and readability of the questionnaire. The pilot included pharmacists from diverse work settings (hospital, community, industry, and academia) with varying levels of professional experience (two with 0–5 years, 10 with 6–10 years, four with 11–15 years, two with 16–20 years, and four with more than 20 years) and different educational qualifications (three diploma holders, six B.Pharm, five PharmD, and six Master's). Feedback obtained from the pilot study, which incorporated a test-retest approach, led to modifications that enhanced the instrument's usability and contextual appropriateness. Following validation, data collection was performed electronically with the support of trained data collectors.

#### Questionnaire contents

Sociodemographic Characteristics: this section consisted of six items that gathered background data, including age, sex, highest academic qualification, years of professional experience, primary practice setting, and prior formal training in DDI management.

Knowledge of DDIs: this domain included 12 multiple-choice questions evaluating the pharmacists' ability to recognize clinically significant DDI pairs. Examples included warfarin with aspirin, simvastatin with clarithromycin, SSRIs with MAOIs, ACE inhibitors with potassium-sparing diuretics, nitroglycerin with sildenafil, amiodarone with atorvastatin, and digoxin with verapamil. Other key interactions assessed were lithium with ACE inhibitors, levodopa with metoclopramide, methotrexate with dapsone, carbamazepine with acetaminophen, and digoxin with furosemide—chosen for their high clinical importance and potential for serious adverse outcomes.

Attitudes toward DDIs: ten items explored pharmacists' perceptions and confidence regarding DDI management. This included attitudes toward using DDI-checking tools, interdisciplinary collaboration, patient counseling, and the significance of DDIs in pharmacy education and ongoing professional development.

Practices in DDI management: this section assessed practical behaviors through six questions focused on the routine use of interaction-checking systems during dispensing, engagement in patient education, participation in continuing education programs, and monitoring of patients on high-risk medications.

Barriers to effective DDI management: ten items identified common obstacles hindering effective DDI management, such as lack of access to up-to-date DDI databases (e.g., Lexicomp, Micromedex), time constraints, challenges in interpreting complex interactions, insufficient institutional support for training, limited interprofessional communication, lack of localized guidelines, and inadequate clinical decision support tools.

This validated questionnaire provided a robust framework for evaluating pharmacists' competencies and the systemic barriers encountered in the management of DDIs within the Saudi Arabian healthcare setting.

### Data collection

Data were collected electronically using the validated questionnaire, developed as a Google Form. A QR code linking to the form was shared at participants' workplaces to facilitate access. Trained authors and researchers serving as data collectors were present at the sites to clarify any ambiguities or questions from participants, ensuring accurate and complete responses. This method allowed standardized, reliable, and efficient collection of data across diverse pharmacy practice settings in Riyadh, Saudi Arabia.

#### Sample size

According to the Healthcare Establishments and Workforce Statistics Publication 2023 issued by the General Authority for Statistics (GASTAT), the total number of licensed pharmacists in Saudi Arabia was 36,810 in 2023 (26). Although region-specific data for Riyadh were not separately reported, Riyadh—being the capital and the most populous region—hosts a substantial proportion of the national pharmacist workforce. The minimum required sample size was calculated using the standard formula for estimating proportions in finite populations, assuming a 95% confidence level, a 5% margin of error, and a response distribution of 50%. The calculated minimum sample size was 370 pharmacists. A final sample size of 409 participants was achieved, ensuring 80% statistical power to detect significant differences at a 5% significance level, thereby enhancing the generalizability and robustness of the findings.

### Data analysis

Data were analyzed using Statistical Package for the Social Sciences [SPSS version 25 (IBM Corp., Armonk, NY, USA)]. Descriptive statistics summarized participants' demographic characteristics and their scores on knowledge, attitudes, practices, and perceived barriers.

In the knowledge domain, each correct response was scored as ‘1' and incorrect responses as ‘0'. The total possible score was 12, with a mean score of 8.04 across all 409 participants. Knowledge was categorized as adequate (≥8.04) or inadequate (< 8.04) (27).

For the attitude domain, responses were rated on a 5-point Likert scale from 1 (strongly disagree/not confident at all) to 5 (strongly agree/very confident). The mean attitude score was 34.37, with participants scoring ≥34.37 classified as having positive attitudes, and those scoring below as having negative attitudes.

The practice domain was rated on a 5-point Likert scale, where 1 corresponded to “never” and 5 to “always”. The mean practice score was 18; scores ≥18 indicated effective practice, while scores < 18 indicated ineffective practice.

For perceived barriers, items were rated using appropriate Likert scales depending on the question format (1–5: never to always, 1–5: strongly agree to strongly disagree, or 1–4: not at all to a large extent). The mean barrier score was 30.55; scores ≥30.55 reflected high perceived barriers, while scores below this threshold indicated low perceived barriers.

Overall, all categorizations were based on cohort mean scores, ensuring consistency across knowledge, attitudes, practices, and barriers (27).

Inferential analysis included binary logistic regression to identify significant predictors of knowledge, attitudes, practices, and barriers, adjusting for relevant sociodemographic factors. Chi-square tests were used to assess associations between categorical variables. Additionally, Pearson's correlation analysis was performed to explore relationships among knowledge, attitudes, practices, and perceived barriers. A *P* value < 0.05 was considered statistically significant.

### Ethical considerations

The study was approved by the Institutional Review Board (IRB) of the University (IRB24–102). All procedures adhered to the ethical principles of the Declaration of Helsinki and complied with the Strengthening the Reporting of Observational Studies in Epidemiology (STROBE) guidelines (https://www.strobe-statement.org/). Informed consent was obtained from all participants, who were assured of confidentiality, anonymity, and voluntary participation, with the freedom to withdraw at any stage without penalty.

## Results

### Demographic characteristics of the participants

Table 1 presents the demographic characteristics of the study participants, including their sex, age, educational background, professional experience, work setting, and formal training on drug interactions. The data revealed that most of the participants were male (63.8%) and aged between 30–39 years (40.8%). In terms of educational qualifications, most participants held a Bachelor of Pharmacy (B.Pharm; 45.2%), with a smaller portion having a Doctor of Pharmacy (Pharm.D.; 31.5%) or a Master's degree (16.1%). With respect to professional experience, most participants had 0–5 years of experience (35.2%). Most worked in hospital pharmacy settings (45.2%), followed by community pharmacies (31.3%). Almost half of the participants (49.4%) reported having formal training on drug interactions.

**Table 1:** Demographic characteristics of the participants.

**Characteristics**	**Variables**	**Frequency**	**Percentage**
Sex	Male	261	63.8
	Female	148	36.2
Age (years)	20–29	145	35.5
	30–39	167	40.8
	40–49	59	14.4
	50–59	31	7.6
	60 and above	7	1.7
Highest qualification	Diploma in Pharmacy	17	4.2
	Bachelor of Pharmacy (B. Pharm)	185	45.2
	Doctor of Pharmacy (Pharm.D)	129	31.5
	Master's Degree	66	16.1
	Ph.D.	10	8.0
Experience as pharmacist	0–5 years	144	35.2
	6–10 years	101	24.7
	11–15 years	96	23.5
	16–20 years	50	12.2
	More than 20 years	18	4.4
Primary work setting	Hospital pharmacy	185	45.2
	Community pharmacy	128	31.3
	Industrial pharmacy	34	8.3
	Academia	45	11.0
	Others	17	4.2
Formal training on drug interaction	Yes	202	49.4
	No	207	50.6

#### Status of knowledge, attitudes, practices, and barriers to drug–drug interactions

Table 2 summarizes participants' knowledge, attitudes, practices, and perceived barriers related to drug–drug interactions. Just over half of the participants demonstrated adequate knowledge (55.7%), while 44.0% had inadequate knowledge. Negative attitudes toward DDIs predominated (63.3%), with only 36.7% reporting positive attitudes. In terms of practice, most participants (71.4%) reported ineffective DDI management, whereas 28.6% indicated effective practices. More than half of the respondents (53.8%) perceived high barriers to effective DDI management, while 46.2% reported low barriers.

**Table 2:** Knowledge, attitudes, practices, and barriers to drug–drug interactions.

**Outcomes**	**Variables**	**Frequency**	**Percent**
Knowledge	In-adequate	180	44.0
	Adequate	228	55.7
Attitude	Negative	259	63.3
	Positive	150	36.7
Practice	In-effective	292	71.4
	Effective	117	28.6
Barriers	High	220	53.8
	Low	189	46.2

Overall, pharmacists exhibited moderate knowledge of clinically significant DDIs, with higher correct response rates for interactions associated with severe adverse outcomes, such as SSRIs with MAOIs, nitroglycerin with sildenafil, lithium with ACE inhibitors, and levodopa with metoclopramide. Lower correct responses were observed for interactions involving digoxin with furosemide and methotrexate with dapsone, indicating specific knowledge gaps (Table 3).

**Table 3:** Frequency distribution of knowledge domain.

**Number**	**Items**	**Incorrect response**		**Correct response**	
		**Frequency**	**Percentage**	**Frequency**	**Percentage**
1	Bleeding risk with warfarin + aspirin	120	29.3	289	70.5
2	Myopathy/rhabdomyolysis risk with simvastatin + clarithromycin	129	31.5	280	68.3
3	Serotonin syndrome risk with SSRIs + MAOIs	95	23.2	314	76.6
4	Hyperkalemia risk with ACE inhibitors + potassium-sparing diuretics	140	34.1	269	65.6
5	Severe hypotension with nitroglycerin + sildenafil	110	26.8	299	72.9
6	Myopathy risk with amiodarone + atorvastatin	150	36.6	259	63.2
7	Bradycardia with digoxin + verapamil	165	40.2	244	59.5
8	Lithium toxicity with lithium + ACE inhibitors	105	25.6	304	74.1
9	Exacerbation of Parkinson's symptoms with levodopa + metoclopramide	99	24.1	310	75.6
10	Hematologic toxicity with methotrexate + dapsone	168	41.0	241	58.8
11	Hepatotoxicity with carbamazepine + acetaminophen	125	30.5	284	69.3
12	Hypokalemia-induced digoxin toxicity with digoxin + furosemide	211	51.5	198	48.3

Attitudinal responses reflected cautious confidence, with many participants selecting neutral options regarding their understanding of DDIs and confidence in professional communication and patient counseling. However, strong agreement was noted on the importance of pharmacists' roles in patient education, continuous knowledge updating, inclusion of DDI management in undergraduate curricula, mandatory continuing education, and the usefulness of real-time clinical decision support systems (Table 4).

**Table 4:** Frequency distribution of attitude domain.

**Number**	**Items**	**Variables**	**Frequency**	**Percentage**
1	To what extent do you feel confident in your understanding of drug-drug interactions?	Not confident at all	25	6.1
		Slightly confident	138	33.7
		No comment	117	28.5
		Moderately confident	68	16.6
		Very confident	61	14.9
2	Do you feel confident discussing drug interactions with other healthcare professionals (e.g., physicians, nurses)?	Not confident at all	43	10.5
		Slightly confident	115	28.0
		No comment	129	31.5
		Moderately confident	53	12.9
		Very confident	69	16.8
3	How confident are you in counseling patients on the potential risks of drug interactions?	Not confident at all	39	9.5
		Slightly confident	113	27.6
		No comment	137	33.4
		Moderately confident	51	12.4
		Very confident	69	16.8
4	How confident are you to be involved in multidisciplinary healthcare teams to manage drug interactions?	Not confident at all	38	9.3
		Slightly confident	111	27.1
		No comment	136	33.2
		Moderately confident	61	14.9
		Very confident	63	15.4
5	Do you believe that you have sufficient access to resources (e.g., drug interaction databases) to identify potential drug interactions in your practice?	Strongly disagree	45	11.0
		Disagree	27	6.6
		Neutral	129	31.5
		Agree	111	27.1
		Strongly agree	97	23.7
6	Do you think drug interaction management should be a mandatory component of continuing education for pharmacists?	Strongly disagree	19	4.6
		Disagree	12	2.9
		Neutral	144	35.1
		Agree	108	26.3
		Strongly agree	126	30.7
7	Do you believe that pharmacists play a critical role in educating patients about drug interactions?	Strongly disagree	25	6.1
		Disagree	10	2.4
		Neutral	115	28.0
		Agree	133	32.4
		Strongly agree	126	30.7
8	Do you think there should be more emphasis on drug interaction management in undergraduate pharmacy education?	Strongly disagree	17	4.1
		Disagree	15	3.7
		Neutral	120	29.3
		Agree	129	31.5
		Strongly agree	128	31.2
9	Do you believe that pharmacists should continuously update their knowledge of drug interactions?	Strongly disagree	20	4.9
		Disagree	16	3.9
		Neutral	126	30.7
		Agree	111	27.1
		Strongly agree	136	33.2
10	Do you believe that integrated clinical decision support system for detecting drug interactions in real time is helpful?	Strongly disagree	21	5.1
		Disagree	10	2.4
		Neutral	135	32.9
		Agree	121	29.5
		Strongly agree	122	29.8

Practice patterns suggested inconsistent application of DDI management in routine care. A substantial proportion of respondents selected neutral responses for activities such as checking interactions, counseling patients, using interaction-checking tools, monitoring high-risk medications, and attending training programs, highlighting a gap between knowledge, attitudes, and actual practice (Table 5).

**Table 5:** Frequency distribution of practice domain.

**Number**	**Items**	**Variables**	**Frequency**	**Percentage**
1	How often do you check for drug-drug interactions when dispensing prescriptions?	Never	25	6.1
		Rarely	64	15.6
		Neutral	200	48.8
		Sometimes	72	17.6
		Always	48	11.7
2	Do you routinely counsel patients about the risks of drug interactions?	Never	42	10.2
		Rarely	50	12.2
		Neutral	200	48.8
		Sometimes	64	15.6
		Always	53	12.9
3	How frequently do you use drug interaction checker tools or software in your practice?	Never	34	8.3
		Rarely	57	13.9
		Neutral	204	49.8
		Sometimes	57	13.9
		Always	57	13.9
4	Have you ever encountered a drug interaction that caused a significant adverse event in a patient?	Never	65	15.9
		Rarely	64	15.6
		Neutral	183	44.6
		Sometimes	53	12.9
		Always	44	10.7
5	How often do you attend training or workshops on drug interactions?	Never	74	18.0
		Rarely	53	12.9
		Neutral	191	46.6
		Sometimes	57	13.9
		Always	34	8.3
6	Do you routinely monitor patients with high-risk medications for potential interactions?	Never	44	10.7
		Rarely	61	14.9
		Neutral	212	51.7
		Sometimes	41	10.0
		Always	51	12.4
		Never	44	10.7

Several systemic barriers were identified, including limited access to updated databases, heavy workload, time constraints, complexity of available resources, and insufficient institutional support, all of which moderately to substantially affected pharmacists' ability to manage DDIs effectively (Table 6).

**Table 6:** Frequency distribution of barrier domain.

**Number**	**Items**	**Variables**	**Frequency**	**Percentage**
1	How often do you have no access to updated databases or resources for checking drug-drug interactions (e.g., Lexicomp, Micromedex, etc.)	Never	30	7.3
		Rarely	59	14.4
		Sometimes	201	49.0
		Often	62	15.1
		Always	57	13.9
2	How often do you feel that a heavy workload interferes with your ability to stay updated on new drug interactions?	Never	28	6.8
		Rarely	51	12.4
		Sometimes	171	41.7
		Often	82	20.0
		Always	77	18.8
3	How often do you face difficulty in interpreting information regarding drug-drug interactions due to the complexity of available resources?	Never	44	10.7
		Rarely	50	12.2
		Sometimes	175	42.7
		Often	89	21.7
		Always	51	12.4
4	How often do you rely solely on personal experience rather than official resources when identifying drug-drug interactions due to lack of time or resources?	Never	34	8.3
		Rarely	52	12.7
		Sometimes	196	47.8
		Often	81	19.8
		Always	46	11.2
5	The training or continuing education provided by your employer on the topic of drug-drug interactions is adequate.	Strongly agree	41	10.0
		Agree	36	8.8
		Neutral	167	40.7
		Disagree	102	24.9
		Strongly disagree	63	15.4
6	Do you believe the collaboration with other healthcare professionals (e.g., physicians) affects your ability to effectively manage drug-drug interactions?	Strongly agree	12	2.9
		Agree	15	3.7
		Neutral	144	35.1
		Disagree	122	29.8
		Strongly disagree	116	28.3
7	What level of support do you receive from your pharmacy management to attend workshops or seminars focused on drug-drug interaction management?	Strongly agree	35	8.5
		Agree	31	7.6
		Neutral	169	41.2
		Disagree	96	23.4
		Strongly disagree	78	19.0
8	To what extent do you feel that a formal clinical guideline specific to drug-drug interactions in your region/country is a barrier to your knowledge?	Not at all	83	20.2
		To a small extent	99	24.1
		To a moderate extent	162	39.5
		To a large extent	65	15.9
9	To what extent do time constraints during your workday prevent you from thoroughly checking for potential drug-drug interactions?	Not at all	75	18.3
		To a small extent	126	30.7
		To a moderate extent	157	38.3
		To a large extent	51	12.4
10	How much does the unavailability of technology (e.g., clinical decision support systems) in your workplace impact your ability to identify and manage drug-drug interactions?	Not at all	94	22.9
		To a small extent	96	23.4
		To a moderate extent	153	37.3
		To a large extent	66	16.1

#### Associations between sociodemographic characteristics and participants' knowledge and attitudes

Table 7 shows significant associations between participants' knowledge of drug–drug interactions and sociodemographic factors. Female participants had higher adequate knowledge (65%) than males (51%, *P* = 0.008). Knowledge increased with age, from 47% in the 30–39 years group to 97% in those 50–59 years and 100% in participants ≥60 years (*P* = 0.000). Educational level influenced knowledge, with master's degree holders showing the highest adequate knowledge (85%), followed by Ph.D. holders (58%, *P* = 0.001). Experience also mattered: pharmacists with 16–20 years (76%) and >20 years (89%) of experience had higher knowledge (*P* = 0.001). Work setting affected knowledge, with academics scoring highest (91%) compared with community (33%) and industrial pharmacists (18%, *P* = 0.001). Formal training did not significantly impact knowledge (*P* = 0.643).

**Table 7:** Comparison of knowledge and attitudes with sociodemographic characteristics.

**Characteristics**	**Variables**	**Knowledge**, ***n*** **(%)**			**Attitude**, ***n*** **(%)**		
		**In-adequate**	**Adequate**	***P*** **value**	**Negative**	**Positive**	***P*** **value**
Sex	Male	128 (49)	133 (51)	**0.008**	157 (60)	104 (40)	0.077
	Female	52 (35)	95 (65)		102 (69)	46 (31)	
Age (years)	20–29	74 (51)	71 (49)	**0.000**	100 (69)	45 (31)	**0.000**
	30–39	88 (53)	78 (47)		88 (53)	79 (47)	
	40–49	17 (29)	42 (71)		35 (59)	24 (41)	
	50–59	1 (3)	30 (97)		29 (94)	2 (6)	
	60 and above	0 (0)	7 (100)		7 (100)	0	
Highest qualification	Diploma in pharmacy	16 (94)	1 (6)	**0.001**	9 (53)	8 (47)	**0.021**
	Bachelor of pharmacy (B. Pharm)	91 (49)	94 (51)		113 (61)	72 (39)	
	Doctor of pharmacy (Pharm.D)	58 (45)	70 (55)		75 (58)	54 (42)	
	Master's degree	10 (15)	56 (85)		53 (80)	13 (20)	
	Ph.D.	5 (42)	7 (58)		9 (75)	3 (25)	
Experience as pharmacist	0–5 years	70 (49)	74 (51)	**0.001**	98 (68)	46 (32)	**0.030**
	6–10 years	52 (52)	48 (48)		54 (54)	47 (47)	
	11–15 years	44 (46)	52 (54)		56 (58)	40 (42)	
	16–20 years	12 (24)	38 (76)		38 (76)	12 (24)	
	More than 20 years	2 (11)	16 (89)		13 (72)	5 (28)	
Primary work setting	Hospital pharmacy	62 (34)	123 (66)	**0.001**	112 (61)	73 (39)	**0.001**
	Community pharmacy	86 (67)	42 (33)		77 (60)	51 (40)	
	Industrial pharmacy	27 (82)	6 (18)		13 (38)	21 (62)	
	Academia	4 (9)	41 (91)		42 (93)	3 (7)	
	Others	1 (6)	16 (94)		15 (88)	2 (12)	
Formal training on DDIs	Yes	91 (45)	110 (55)	0.643	107 (53)	95 (47)	**0.001**
	No	89 (43)	118 (57)		152 (73)	55 (27)	

Bold *P* value correspond to *P* < 0.05 and are considered statistically significant.

Regarding attitudes, age strongly influenced participants' outlook: younger participants (20–29 years) were most negative (69%), whereas older participants (50–59 years, 6%; ≥60 years, 0%) were largely positive (*P* = 0.000). Sex had no significant effect (*P* = 0.077). Participants with higher qualifications and greater experience tended to show more negative attitudes, particularly those with master's degrees (80%) or Ph.D.s (75%, *P* = 0.021) and 16–20 (76%) or >20 years of experience (72%, *P* = 0.030). Work setting also mattered, with academics (93%) and hospital pharmacists (61%) showing more negative attitudes than community (40%) and industrial pharmacists (38%, *P* = 0.001). Formal training significantly improved attitudes, with 47% of trained participants exhibiting positive attitudes vs. 27% of untrained participants (*P* = 0.001). These findings indicate that higher education, extensive experience, and formal training are linked to better knowledge and more positive attitudes toward managing drug–drug interactions.

#### Associations between sociodemographic characteristics and practices and barriers to DDIs

Table 8 presents associations between sociodemographic characteristics and practices in managing drug–drug interactions. Sex significantly influenced practice, with males showing more effective practice (33%) than females (20%, *P* = 0.002). Younger participants (20–29 years) had the lowest effective practice (21%), while 30–39 years showed higher levels (47%, *P* = 0.000). Pharmacists with 6–10 years of experience had the highest effective practice (50%), compared with 16–20 years (6%, *P* = 0.001). Work setting also mattered: industrial pharmacists demonstrated the highest effective practice (53%), while academics had the lowest (7%, *P* = 0.001). Formal training significantly improved practice, with trained participants achieving 44% effectiveness vs. 13% for untrained (*P* = 0.001).

**Table 8:** Comparison of practices and barriers with sociodemographic characteristics.

**Characteristics**	**Variables**	**Practice**, ***n*** **(%)**		***P* value**	**Barriers**, ***n*** **(%)**		***P* value**
		**In-effective**	**Effective**		**High**	**Low**	
Sex	Male	173 (66)	88 (33)	**0.002**	140 (54)	121 (46)	0.936
	Female	119 (80)	29 (20)		80 (54)	68 (46)	
Age (years)	20–29	114 (79)	31 (21)	**0.000**	98 (68)	47 (32)	**0.000**
	30–39	89 (53)	78 (47)		65 (39)	102 (61)	
	40–49	52 (88)	7 (12)		27 (46)	32 (54)	
	50–59	30 (97)	1 (3)		23 (74)	8 (26)	
	60 and above	7 (100)	0 (0)		7 (100)	0	
Highest qualification location marital status	Diploma in Pharmacy	12 (71)	5 (29)	0.160	11 (65)	6 (35)	**0.001**
	Bachelor of Pharmacy (B.Pharm)	136 (74)	49 (27)		79 (43)	106 (57)	
	Doctor of Pharmacy (Pharm.D)	83 (64)	46 (36)		76 (59)	53 (41)	
	Master's Degree	50 (76)	16 (24)		42 (64)	24 (36)	
	Ph.D.	11 (92)	1 (8)		12 (100)	0	
Experience as a pharmacist	0–5 years	110 (76)	34 (24)	**0.001**	103 (72)	41 (29)	**0.001**
	6–10 years	50 (50)	51 (50)		40 (40)	61 (60)	
	11–15 years	70 (73)	26 (27)		34 (35)	62 (65)	
	16–20 years	47 (94)	3 (6)		30 (60)	20 (40)	
	More than 20 years	15 (83)	3 (17)		13 (72)	5 (28)	
Primary work setting	Hospital pharmacy	122 (66)	63 (34)	**0.001**	84 (45)	101 (55)	**0.001**
	Community pharmacy	98 (77)	30 (23)		62 (48)	66 (52)	
	Industrial Pharmacy	16 (47)	18 (53)		17 (50)	17 (50)	
	Academia	42 (93)	3 (7)		42 (93)	3 (7)	
	others	14 (82)	3 (18)		15 (88)	2 (12)	
Formal training on DDIs	Yes	113 (56)	89 (44)	**0.001**	99 (49)	103 (51)	0.055
	No	179 (87)	28 (13)		121 (59)	86 (41)	

Bold *P* value correspond to *P* < 0.05 and are considered statistically significant.

Regarding perceived barriers, age had a strong effect, with 20–29-year-olds reporting the most barriers (68%, *P* = 0.000). Educational level influenced barriers, with Ph.D. holders reporting the highest (100%) and Master's (36%) and Pharm.D. participants (41%) reporting moderate levels (*P* = 0.001). Experience affected barriers, with >20 years reporting the highest (72%) and 16–20 years fewer (60%, *P* = 0.001). Work setting showed similar trends, with academics reporting the highest barriers (93%) and community (52%) and hospital pharmacists (55%) lower (*P* = 0.001). Sex and formal training did not significantly impact barriers (*P* = 0.936 and *P* = 0.055, respectively), though training was associated with more balanced barrier perception.

#### Factors influencing the knowledge and attitudes of the participants toward DDIs

Logistic regression analysis showed that age, sex, and highest qualification were significant predictors of pharmacists' knowledge of drug–drug interactions. Knowledge increased with age (*P* < 0.001, OR = 2.890), and male pharmacists demonstrated higher knowledge levels than females (*P* = 0.003, OR = 1.994). Higher academic qualifications were also positively associated with knowledge (*P* = 0.001, OR = 1.617). In contrast, years of experience, primary work setting, and formal DDI training were not significantly associated with knowledge (Table 9). Attitudes toward DDIs were significantly influenced by age, qualification, and formal training. Younger pharmacists exhibited more positive attitudes (*P* = 0.040, OR = 0.618), as did those with higher qualifications (*P* = 0.028, OR = 0.740) and those who had received formal DDI training (*P* < 0.001, OR = 2.590). Sex, professional experience, and work setting were not significant predictors of attitude, suggesting a limited role in shaping perceptions toward DDIs (Table 9).

**Table 9:** Factors influencing knowledge and attitudes toward drug-drug interactions.

**Independent variables**	**Knowledge**				**Attitude**			
	***P*** **Value**	**Odds ratio**	**95% Confidence interval for the odds ratio**		***P*** **Value**	**Odds ratio**	**95% Confidence interval for the odds ratio**	
			**Lower Bound**	**Upper Bound**			**Lower Bound**	**Upper Bound**
Age	**0.000**	2.890	1.751	4.770	**0.040**	0.618	0.390	0.978
Sex	**0.003**	1.994	1.259	3.158	0.114	0.690	0.436	1.093
Highest qualification	**0.001**	1.617	1.220	2.144	**0.028**	0.740	0.566	0.968
Experience as a pharmacist	0.064	0.710	0.494	1.020	0.080	1.368	0.963	1.944
Primary work setting	0.431	1.088	0.882	1.341	0.178	0.862	0.695	1.070
Formal training on drug interactions	0.358	0.804	0.504	1.281	**0.000**	2.590	1.625	4.126

Bold *P* value correspond to *P* < 0.05 and are considered statistically significant.

#### Factors influencing practices and barriers to drug–drug interactions

Regression analysis identified sex and formal DDI training as significant predictors of pharmacists' practice. Female pharmacists reported better practices than males (*P* < 0.001, OR = 0.381), while formal training was the strongest determinant of appropriate practice (*P* < 0.001, OR = 5.467). Age, highest qualification, years of experience, and work setting were not significantly associated with practice (Table 10). Perceived barriers to DDI management were significantly influenced by age, qualification, experience, and work setting. Younger pharmacists (*P* = 0.024, OR = 0.593) and those with higher qualifications (*P* = 0.004, OR = 0.675) reported fewer barriers, whereas more experienced pharmacists perceived more obstacles (*P* < 0.001, OR = 1.933). Work setting was also a significant factor (*P* < 0.001, OR = 0.621). Sex and formal training did not show significant associations with perceived barriers (Table 10).

**Table 10:** Factors influencing practice and barriers to DDIs.

**Independent variables**	**Practice**				**Barriers**			
	***P*** **Value**	**Odds ratio**	**95% Confidence Interval for the Odds Ratio**		***P*** **Value**	**Odds ratio**	**95% Confidence Interval for the Odds Ratio**	
			**Lower Bound**	**Upper Bound**			**Lower Bound**	**Upper Bound**
Age	0.078	0.621	0.365	1.054	**0.024**	0.593	0.376	0.935
Sex	**0.000**	0.381	0.221	0.655	0.544	1.149	0.733	1.801
Highest qualification	0.553	0.917	0.687	1.223	**0.004**	0.675	0.518	0.881
Experience as a pharmacist	0.796	1.053	0.713	1.554	**0.000**	1.933	1.337	2.795
Primary work setting	0.683	0.952	0.750	1.207	**0.000**	0.621	0.497	0.776
Formal training on drug interactions	**0.000**	5.467	3.168	9.436	0.201	1.348	0.853	2.130

Bold *P* value correspond to *P* < 0.05 and are considered statistically significant.

#### Correlations among outcome variables

Table 11 presents the Pearson correlation analysis among knowledge, attitude, practice, and barrier levels. Knowledge level showed no significant correlation with attitude (*r* = −0.054, *P* = 0.277), practice (*r* = 0.035, *P* = 0.484), or barrier level (*r* = −0.001, *P* = 0.991), indicating that DDI-related knowledge was not directly associated with other outcome domains. In contrast, attitude level demonstrated a significant positive correlation with practice (*r* = 0.439, *P* < 0.001) and barrier level (*r* = 0.312, *P* < 0.001). Similarly, practice level was positively correlated with attitude (*r* = 0.439, *P* < 0.001) and barrier level (*r* = 0.336, *P* < 0.001). Barrier level also showed significant positive correlations with attitude (*r* = 0.312, *P* < 0.001) and practice (*r* = 0.336, *P* < 0.001). Overall, the findings indicate a strong interrelationship between attitudes and practices, with fewer perceived barriers associated with more positive attitudes and better practices. However, knowledge level did not demonstrate significant associations with attitudes, practices, or barriers in this study.

**Table 11:** Correlation analysis for outcome variables.

**Outcome variables**		**Knowledge Level**	**Attitude Level**	**Practice Level**	**Barrier Level**
Knowledge level	Pearson correlation	1	−0.054	0.035	−0.001
	Sig. (2-tailed)	—	0.277	0.484	0.991
Attitude level	Pearson correlation	−0.054	1	0.439^**^	0.312^**^
	Sig. (2-tailed)	0.277	—	**0.000**	**0.000**
Practice level	Pearson correlation	0.035	0.439^**^	1	0.336^**^
	Sig. (2-tailed)	0.484	**0.000**	—	**0.000**
Barrier level	Pearson correlation	−0.001	0.312^**^	0.336^**^	1
	Sig. (2-tailed)	0.991	**0.000**	**0.000**	—

^**^Correlation is significant at the 0.01 level (2-tailed); Bold Sig. (2-tailed) correspond to *P* < 0.05 and are considered statistically significant.

## Discussion

This study provides an in-depth evaluation of the knowledge, attitudes, practices, and perceived barriers related to drug–drug interaction (DDI) management among pharmacists in Riyadh, Saudi Arabia. As key stakeholders in ensuring medication safety, pharmacists occupy a frontline position in preventing harmful interactions and promoting optimal pharmacotherapy. The findings underscore the influence of demographic and professional variables—particularly sex, age, experience, and formal training—on pharmacists' readiness to manage DDIs effectively. Importantly, the observed gaps between knowledge, attitudes, and practices highlight critical translational challenges that warrant focused educational and policy-level interventions.

The participant profile indicated a predominance of male respondents (63.8%), with most pharmacists aged 30–39 years (40.8%). The overall mean knowledge score (8.01 ± 3.1), equating to 56% of the total possible score, reveals a moderate level of understanding with considerable variation. While mean-based categorization allowed standardized comparisons across domains, this approach may mask clinically meaningful distinctions among pharmacists with borderline or uneven competencies. However, it remains a commonly applied method in KAP studies and provides a pragmatic framework for population-level assessment. Future analyses could incorporate sensitivity or percentile-based approaches to better capture nuanced performance differences. This knowledge gap aligns with earlier Saudi-based findings, where only 12.2% of pharmacists demonstrated excellent DDI-related knowledge (28). Such deficiencies, if unaddressed, could pose significant threats to patient safety due to missed or mismanaged interactions.

Binary logistic regression analysis identified significant predictors of knowledge. Female pharmacists demonstrated higher knowledge than males (*P* = 0.003, OR = 1.994), supporting previous findings that suggest sex-based variations in professional engagement (29, 30). This contrasts with a previous Saudi study that found no significant sex difference (24), which may be due to sample composition differences. Knowledge was also significantly associated with age (*P* < 0.001, OR = 2.890), supporting literature that links clinical experience with improved pharmacological understanding (31). Additionally, pharmacists with higher academic qualifications exhibited greater knowledge (*P* = 0.001, OR = 1.617), particularly those in academic or teaching environments, likely due to regular engagement with evidence-based resources (32).

Attitudinal findings were similarly influenced by demographic and professional factors. Younger pharmacists showed more favorable attitudes (*P* = 0.040, OR = 0.618), possibly reflecting greater openness to continuous learning and newer technologies. Notably, pharmacists with higher qualifications were also more likely to express positive attitudes (*P* = 0.028, OR = 0.740). Furthermore, those with formal DDI training were significantly more likely to hold favorable attitudes (*P* < 0.001, OR = 2.590), reinforcing the value of structured educational programs in shaping professional outlooks. The discrepancy between moderate knowledge levels and relatively positive attitudes suggests the presence of social desirability bias, a common limitation of self-reported surveys, where respondents may overestimate favorable perceptions despite knowledge gaps.

In examining factors that influence practice behaviors, formal training on DDIs emerged as the strongest predictor (*P* < 0.001, OR = 5.467), indicating that pharmacists who had received relevant training were over five times more likely to engage in effective practice. Sex was also significant, with female pharmacists more likely to report better practices (*P* < 0.001, OR = 0.381), despite the discussion noting higher male performance. This updated result suggests a need to reassess assumptions about the sex-practice relationship, potentially exploring external factors such as practice setting, role responsibilities, or confidence levels. This divergence between knowledge, attitudes, and practice reinforces the likelihood of recall and reporting biases and underscores the need for objective practice-based assessments in future studies. In contrast, experience, qualification, and work setting did not significantly influence practice, suggesting that training plays a more pivotal role than years of service or educational attainment in determining DDI-related behaviors.

Barriers to optimal DDI management were reported across the cohort, but specific trends emerged. Younger pharmacists reported fewer perceived barriers (*P* = 0.024, OR = 0.593), while those with higher qualifications also noted fewer obstacles (*P* = 0.004, OR = 0.675). Pharmacists with more years of experience, on the other hand, reported significantly more barriers (*P* < 0.001, OR = 1.933), which may reflect greater awareness of systemic limitations or increased workload responsibilities. Interestingly, work setting also influenced barrier perception (*P* < 0.001, OR = 0.621), suggesting that institutional environments play a role in shaping perceived constraints. Despite its benefits in improving practice and attitudes, formal training did not significantly reduce perceived barriers (*P* = 0.201), indicating that many challenges—such as lack of access to tools or heavy workloads—are structural and not easily addressed by education alone (33).

A particularly noteworthy result was the moderate positive correlation between attitudes and practices (*r* = 0.439; *P* < 0.001), suggesting that pharmacists with favorable attitudes are more likely to engage in effective DDI-related practices. This is consistent with previous research from Lebanon, which found that positive perceptions of clinical pharmacy services were predictive of proactive professional behavior (34). Nevertheless, the imperfect correlation suggests that positive attitudes alone are insufficient and must be supported by enabling systems, policies, and tools to translate intent into practice.

Recent evidence supports that computerized clinical decision support systems (CDSS) integrated into prescribing workflows are associated with reductions in medication errors and adverse drug events, though benefits depend on system design and context (35). However, high rates of alert overrides remain a challenge: a recent meta-analysis found that around 90% of DDI alerts are overridden by clinicians, suggesting that alert fatigue and low specificity are critical obstacles (36). Within the framework of Saudi Arabia's Vision 2030, national digital health transformation initiatives—such as unified electronic health records and e-prescribing platforms—offer a strategic opportunity to deploy optimized, pharmacist-inclusive CDSS solutions. Policymakers should prioritize context-sensitive alert algorithms, tiered severity classifications, and pharmacist-led alert validation to improve adoption and effectiveness.

Interprofessional education (IPE) focused on medication safety has been shown to improve team attitudes, communication, and medication-safety behaviors across professions (37). Embedding DDI-focused IPE modules within undergraduate curricula and continuing professional development aligns with Vision 2030 goals of workforce readiness, patient safety, and quality of care. Such initiatives can strengthen pharmacist–physician collaboration, particularly in managing high-risk polypharmacy.

A scoping review of pharmacy practice in the Middle East identified persistent barriers such as limited continuing professional development, variability in clinical services implementation, and infrastructure constraints—echoing our findings that structural challenges remain beyond individual training (38). Similarly, literature on medication-CDSS adoption highlights the importance of considering human-computer interaction, alert usability, workflow integration, and clinician acceptance (39).

Based on these findings, several practical recommendations can be made to improve DDI management among pharmacists in Saudi Arabia. First, educational programs should be tailored to address specific knowledge and attitudinal gaps, especially targeting younger and less experienced professionals. Second, peer mentorship and experiential learning initiatives can help early-career pharmacists build competence and confidence. Third, formal DDI and pharmacovigilance training should be integrated into continuing professional development frameworks across all pharmacy sectors. Finally, healthcare institutions must address structural barriers by ensuring access to reliable interaction-checking tools, allocating time for professional development, and strengthening decision-support infrastructure. At the policy level, alignment with Vision 2030 requires integrating pharmacist-led medication safety indicators, incentivizing CDSS adoption, and standardizing DDI management protocols across healthcare sectors.

Despite its contributions, this study has limitations. The cross-sectional design limits causal inference, and reliance on self-reported data introduces potential social desirability and recall bias, particularly in attitudes and practices. The exclusive focus on an urban population further limits generalizability, as pharmacists in rural or resource-limited settings may face distinct challenges related to staffing, access to technology, and training opportunities. Therefore, extrapolation beyond similar urban contexts should be undertaken cautiously. Future research should include rural settings, longitudinal designs, objective practice measures, and qualitative approaches to deepen contextual understanding of DDI management.

## Conclusion

This study offers valuable insights into the factors shaping pharmacists' knowledge, attitudes, practices, and perceived barriers in managing drug–drug interactions (DDIs) in Saudi Arabia. Key determinants such as formal training, sex, age, and professional experience were found to significantly influence DDI-related practices. A strong positive correlation between attitudes and practices highlights the importance of motivation and awareness in promoting safe pharmacotherapy. Although formal training improved individual competence, systemic and institutional barriers persisted, highlighting the need for organizational support. Practical recommendations to reduce the burden of DDIs include implementing structured training programs, ensuring access to up-to-date electronic DDI databases, establishing clear institutional protocols and clinical decision-support systems, promoting interdisciplinary collaboration, and conducting regular audits with feedback to reinforce best practices. Empowering pharmacists with the necessary skills, tools, and institutional backing is essential to minimize preventable medication-related harm and enhance patient safety.

## Data Availability

The raw data supporting the conclusions of this article will be made available by the authors, without undue reservation.
