# Supplementary material for: Knowledge, attitudes, practices, and barriers toward drug–drug interaction management among pharmacists in Riyadh, Saudi Arabia: a cross-sectional study
Source: Front Public Health. 2026 Mar 13;14:1727731. doi: 10.3389/fpubh.2026.1727731 (PMC13021610; doi:10.3389/fpubh.2026.1727731)
Supplement: Supplementary file 1 [file Data_Sheet_1.pdf]

Knowledge, Attitudes, Practices, and Barriers Toward Drug–Drug Interaction Management Among Pharmacists in Riyadh, Saudi Arabia: A Cross-Sectional Study

Othman AlOmeir[1], Abdullah Khatim R Alanazi[2], Mulham Ahmed Alnhas[2], Mansour Ahmed Ajarem[2], Zakwan Jamilurehman Hafiz[2], Syed AbdulMuqeet[2], Seham Muqbil Alanazi[3], Majed Sadun Alshammari[4], Hessah khaled ALjanobi[5], Hamoud Alotaibi[6], Deemah Alhamad[6], Nada Aldhahri[6], Majed AlJuaie[7], Syed Mohammed Basheeruddin Asdaq[2,8]*

[1]Department of Pharmacy Practice, College of Pharmacy, Shaqra University, Shaqra 11961, Saudi Arabia, O.k.alomeir@gmail.com
[2]Department of Pharmacy Practice, College of Pharmacy, AlMaarefa University, Dariyah, 13713, Riyadh, Saudi Arabia, sasdaq@gmail.com/sasdag@um.edu.sa (SMBA); 202120443@student.um.edu.sa (AKRA), 192120534@student.um.edu.sa (MAA); 181120235@student.um.edu.sa (MAA); 201120110@student.um.edu.sa (ZJH); 181120034@student.um.edu.sa (SA)
[3]Assistant Consultant, King Abdulaziz Medical City, Riyadh, Saudi Arabia, alanazise5@ngha.med.sa
[4]Department of Pharmacy, King Abdulaziz Medical City, Riyadh, Saudi Arabia, majedalhosaini@gmail.com (MSA)
[5]Department of Nursing, King Abdulaziz Medical City, Riyadh, Saudi Arabia ganobaih@ngha.med.sa
[6]Department of Pharmacy, King Saud University Medical City, Riyadh, Saudi Arabia. hoalotaibi@ksu.edu.sa (H.A), dalhamad@ksu.edu.sa (D.A), naldhari@ksu.edu.sa (N.DH),
[7]Department of Pharmacy, King Khaled Eye Specialist Hospital & Research Center, Riyadh, Saudi Arabia. majed.aljuaie@gmail.com (M.A)
[8]Research Center, Deanship of Scientific Research and Postgraduate Studies, AlMaarefa University, Dariyah, 13713, Riyadh, Saudi Arabia

*** Correspondence:**
Syed Mohammed Basheeruddin Asdaq,
sasdaq@gmail.com

Section 1: Sociodemographic Information

1. **Age**
    - 20–29
    - 30–39
    - 40–49
    - 50–59
    - 60 and above
2. **Gender**
    - Male
    - Female
3. **Highest Qualification**
    - Diploma in Pharmacy
    - Bachelor of Pharmacy (B.Pharm)
    - Doctor of Pharmacy (Pharm.D)
    - Master's Degree
    - Ph.D.
    - Other (Please specify): ________________
4. **Years of Experience as a Pharmacist**
    - 0–5 years
    - 6–10 years
    - 11–15 years
    - 16–20 years
    - More than 20 years
5. **Primary Work Setting**
    - Hospital pharmacy
    - Community pharmacy
    - Industrial Pharmacy
    - Academia
    - Other (Please specify): ________________

6. **Have you received any formal training on drug interactions?**
    - Yes
    - No

Section 2: Knowledge

1. **What is the primary risk when prescribing Warfarin and Aspirin together?**
    a. Risk of thrombosis.
    b. Risk of gastrointestinal bleeding.
    c. Risk of hypertension.
    d. Risk of respiratory distress.

2. **What is the main risk of concurrent use of Simvastatin and Clarithromycin?**
    a. Hyperglycemia.
    b. Myopathy.
    c. Hepatotoxicity.
    d. Renal failure.

3. **Combining SSRIs and MAOIs can result in:**
    a. Serotonin syndrome.
    b. Hypotension.
    c. Hepatotoxicity.
    d. Nephrotoxicity.

4. **What is the primary concern when using ACE inhibitors like Lisinopril with Potassium-sparing diuretics?**
    a. Hypokalemia.
    b. Hyperkalemia.
    c. Hypernatremia.
    d. Hyponatremia.

5. **Nitroglycerin and Sildenafil combined can lead to:**
    a. Severe hypotension.
    b. Severe hypertension.
    c. Tachycardia.
    d. Hyperglycemia.

6. **What is the primary risk of combining Amiodarone and Atorvastatin?**
    a. Myopathy.
    b. Bradycardia.
    c. Hepatotoxicity.
    d. Hyperglycemia.

7. **Co-administration of Digoxin and Verapamil may increase the risk of:**
    a. Bradycardia.
    b. Tachycardia.
    c. Hypoglycemia.
    d. Hepatotoxicity.

8. Combining Lithium and ACE inhibitors like Lisinopril can lead to:

a. Lithium toxicity.
b. GI disturbances.
c. Hyperkalemia.
d. Hypertension.

9. What is the risk associated with concurrent use of Levodopa and Metoclopramide?

a. Exacerbation of Parkinson's symptoms.
b. Severe hypoglycemia.
c. Tachycardia.
d. Hepatotoxicity.

10. A patient on Methotrexate and Dapsone is at increased risk for:

a. Methotrexate toxicity.
b. Hepatotoxicity.
c. Anemia.
d. Renal failure.

11. Carbamazepine and Acetaminophen combination increases the risk of:

a. Hepatotoxicity.
b. Gastrointestinal bleeding.
c. Seizures.
d. Headaches.

12. What is the main concern when combining Digoxin and Furosemide?

a. Hypokalemia.
b. Hyperkalemia.
c. Hyponatremia.
d. Hepatotoxicity.

Section 3: Attitudes

1. To what extent do you feel confident in your understanding of drug-drug interactions?

a) Not confident at all
b) Slightly confident
c) No comment
d) Moderately confident
e) Very confident

2. Do you feel confident discussing drug interactions with other healthcare professionals (e.g., physicians, nurses)?

a) Not confident at all
b) Slightly confident
c) No comment
d) Moderately confident
e) Very confident

3. How confident are you in counseling patients on the potential risks of drug interactions?

a) Not confident at all
b) Slightly confident
c) No comment
d) Moderately confident
e) Very confident

4. How confident are you to be involved in multidisciplinary healthcare teams to manage drug interactions?

a) Not confident at all
b) Slightly confident
c) No comment
d) Moderately confident
e) Very confident

5 Do you believe that you have sufficient access to resources (e.g., drug interaction databases) to identify potential drug interactions in your practice?

a) Strongly disagree
b) Disagree
c) Neutral
d) Agree
e) Strongly agree

6. Do you think drug interaction management should be a mandatory component of continuing education for pharmacists?

a) Strongly disagree
b) Disagree

c) Neutral
d) Agree
e) Strongly agree

7. Do you believe that pharmacists play a critical role in educating patients about drug interactions?
a) Strongly disagree
b) Disagree
c) Neutral
d) Agree
e) Strongly agree

8. Do you think there should be more emphasis on drug interaction management in undergraduate pharmacy education?
a) Strongly disagree
b) Disagree
c) Neutral
d) Agree
e) Strongly agree

9. Do you believe that pharmacists should continuously update their knowledge of drug interactions?
a) Strongly disagree
b) Disagree
c) Neutral
d) Agree
e) Strongly agree

10. Do you believe that integrated clinical decision support system for detecting drug interactions in real time is helpful?
a) Strongly disagree
b) Disagree
c) Neutral
d) Agree
e) Strongly agree

Section 4: Practice

1. How often do you check for drug-drug interactions when dispensing prescriptions?

a) Never
b) Rarely
c) Neutral
d) Sometimes
e) Always

2. Do you routinely counsel patients about the risks of drug interactions?

a) Never
b) Rarely
c) Neutral
d) Sometimes
e) Always

3. How frequently do you use drug interaction checker tools or software in your practice?

a) Never
b) Rarely
c) No comment
d) Sometimes
e) Always

4. Have you ever encountered a drug interaction that caused a significant adverse event in a patient?

a) Never
b) Rarely
c) Neutral
d) Sometimes
e) Always

5. How often do you attend training or workshops on drug interactions?

a) Never
b) Rarely
c) No comment
d) Sometimes
e) Frequently

6. Do you routinely monitor patients with high-risk medications for potential interactions?

a) Never
b) Rarely
c) No comment
d) Sometimes
e) Always

Section 5: Barriers

1. **How often do you have no access to updated databases or resources for checking drug-drug interactions (e.g., Lexicomp, Micromedex, etc.)?**
    a) Never
    b) Rarely
    c) Sometimes
    d) Often
    e) Always

2. **How often do you feel that a heavy workload interferes with your ability to stay updated on new drug interactions?**
    - Never
    - Rarely
    - Sometimes
    - Often
    - Always

3. **How often do you face difficulty in interpreting information regarding drug-drug interactions due to the complexity of available resources?**
    - Never
    - Rarely
    - Sometimes
    - Often
    - Always

4. **How often do you rely solely on personal experience rather than official resources when identifying drug-drug interactions due to lack of time or resources?**
    - Never
    - Rarely
    - Sometimes
    - Often
    - Always

5. **The training or continuing education provided by your employer on the topic of drug-drug interactions is adequate.**
    a) Strongly agree
    b) Agree
    c) Neutral
    d) Disagree
    e) Strongly disagree

6. **Do you believe the collaboration with other healthcare professionals (e.g., physicians) affects your ability to effectively manage drug-drug interactions?**

a) Strongly agree
b) Agree
c) Neutral
d) Disagree
e) Strongly disagree

7. **What level of support do you receive from your pharmacy management to attend workshops or seminars focused on drug-drug interaction management?**
    a) Strongly agree
    b) Agree
    c) Neutral
    d) Disagree
    e) Strongly disagree

8. **To what extent do you feel that a formal clinical guideline specific to drug-drug interactions in your region/country is a barrier to your knowledge?**
    - Not at all
    - To a small extent
    - To a moderate extent
    - To a large extent

9. **To what extent do time constraints during your workday prevent you from thoroughly checking for potential drug-drug interactions?**
    - Not at all
    - To a small extent
    - To a moderate extent
    - To a large extent

10. **How much does the unavailability of technology (e.g., clinical decision support systems) in your workplace impact your ability to identify and manage drug-drug interactions?**
    - Not at all
    - To a small extent
    - To a moderate extent
    - To a large extent
